# High-frequency irreversible electroporation (H-FIRE) for non-thermal ablation without muscle contraction

**DOI:** 10.1186/1475-925X-10-102

**Published:** 2011-11-21

**Authors:** Christopher B Arena, Michael B Sano, John H Rossmeisl, John L Caldwell, Paulo A Garcia, Marissa Nichole Rylander, Rafael V Davalos

**Affiliations:** 1 Bioelectromechanical Systems Lab, Virginia Tech-Wake Forest University School of Biomedical Engineering and Sciences, 330 ICTAS Building (MC0298), Blacksburg, VA. 24061, USA; 2 Neurology and Neurosurgery, Virginia-Maryland Regional College of Veterinary Medicine, Small Animal Clinical Sciences Phase II (MC0442), Blacksburg, VA. 24061, USA; 3 Bioelectromechanical Systems Lab, Virginia Tech Bradley Department of Electrical and Computer Engineering, 330 ICTAS Building (MC0298), Blacksburg, VA. 24061, USA; 4 Tissue Engineering, Nanotechnology, and Cancer Research Lab, Virginia Tech-Wake Forest University School of Biomedical Engineering and Sciences, 335 ICTAS Building (MC0298), Blacksburg, VA. 24061, USA

**Keywords:** Bipolar pulses, Biphasic pulses, Focal ablation, Focal therapy, Heterogeneous tissue, Nerve stimulation, Thermal damage, Electropermeabilization, Electrochemotherapy, nanosecond Pulsed Electric Field

## Abstract

**Background:**

Therapeutic irreversible electroporation (IRE) is an emerging technology for the non-thermal ablation of tumors. The technique involves delivering a series of unipolar electric pulses to permanently destabilize the plasma membrane of cancer cells through an increase in transmembrane potential, which leads to the development of a tissue lesion. Clinically, IRE requires the administration of paralytic agents to prevent muscle contractions during treatment that are associated with the delivery of electric pulses. This study shows that by applying high-frequency, bipolar bursts, muscle contractions can be eliminated during IRE without compromising the non-thermal mechanism of cell death.

**Methods:**

A combination of analytical, numerical, and experimental techniques were performed to investigate high-frequency irreversible electroporation (H-FIRE). A theoretical model for determining transmembrane potential in response to arbitrary electric fields was used to identify optimal burst frequencies and amplitudes for *in vivo *treatments. A finite element model for predicting thermal damage based on the electric field distribution was used to design non-thermal protocols for *in vivo *experiments. H-FIRE was applied to the brain of rats, and muscle contractions were quantified via accelerometers placed at the cervicothoracic junction. MRI and histological evaluation was performed post-operatively to assess ablation.

**Results:**

No visual or tactile evidence of muscle contraction was seen during H-FIRE at 250 kHz or 500 kHz, while all IRE protocols resulted in detectable muscle contractions at the cervicothoracic junction. H-FIRE produced ablative lesions in brain tissue that were characteristic in cellular morphology of non-thermal IRE treatments. Specifically, there was complete uniformity of tissue death within targeted areas, and a sharp transition zone was present between lesioned and normal brain.

**Conclusions:**

H-FIRE is a feasible technique for non-thermal tissue ablation that eliminates muscle contractions seen in IRE treatments performed with unipolar electric pulses. Therefore, it has the potential to be performed clinically without the administration of paralytic agents.

## Background

Irreversible electroporation (IRE) has recently emerged as a non-thermal treatment modality to destroy tumors [1-3] and other non-cancerous pathologies [4-6]. The protocol involves delivering a series of short and intense electric pulses through electrodes inserted directly into, or placed around, the target tissue. The pulses are designed to generate an electric field between the electrodes capable of inducing a rapid buildup of charge across the plasma membrane of cells, commonly referred to as a transmembrane potential (TMP). Once the TMP reaches a critical voltage, it is thought that electrically conductive pores form in the membrane in attempt to prevent permanent damage by shunting current and limiting further TMP rise [7]. If the pulse amplitude and duration are tuned to permit pore resealing, and cell viability is maintained following exposure, the process is categorized as reversible electroporation. This phenomenon has shown great promise in biotechnology and in medicine as a cancer therapy when combined with chemotherapeutic agents (Electrochemotherapy) [8,9] or plasmid DNA (Electrogenetherapy) [10]. IRE results if excess current is applied, and the extent of pore formation is such that the cell cannot recover. Macroscopically, this results in the creation of a tissue lesion without a dependence on thermal processes or the requirement of adjuvant drugs [1,4,11].

Despite requiring electric field strengths on the order of 1000 V/cm, the extent of thermal damage during IRE is negligible due to the short duration of the pulses (on the order of 100 μs) [1,12,13], the low repetition rate of the pulses (on the order of 1 Hz) [14], and the brief treatment time (a few minutes) [14]. These parameters mitigate the associated Joule heating in a majority of the treated tissue, excluding localized regions of elevated electric field adjacent to electrode edges. Due to its inherent non-thermal nature, protein rich tissue components, such as extracellular matrix, are unaffected by IRE. This promotes sparing of sensitive structures, including major blood vessel [5] and nerve [15] architecture. Further, because the mechanism of cell death is non-thermal and relies mainly on the induced TMP, treatment outcomes are not subject to heat sink effects from nearby blood vessels [16]. The induced TMP is predominantly a function of the electric field distribution in the tissue, in addition to cell specific variables. Therefore, knowledge of the field distribution can be used to accurately predict the lesion volume [17,18], which forms with a sharp delineation between treated and unaffected areas in homogenous tissue [4].

In heterogeneous tissues, information on multiple tissue types, including their electrical properties and often times intricate geometrical arrangement complicates treatment planning. For example, subcutaneous tumors treated over the skin non-invasively with IRE using plate electrodes may experience sub-lethal electric fields. This can be attributed to the low conductivity of the skin, which results in a large potential drop across it that increases the likelihood of thermal damage [19]. Up until this point, IRE pulses have been unipolar, meaning that they have a strong DC frequency component (0 Hz). Recently, we have theoretically shown that by delivering high-frequency, bipolar bursts, the impedance barrier of the skin or other epithelial layers can be overcome [20]. Specifically, squarewave bursts with a center frequency around 500 kHz (duration of single polarity equal to one microsecond) produce more homogenous, predictable electric field distributions in a heterogeneous geometry and reduce the potential for thermal damage. This is due to the fact that tissue contains both resistive and capacitive components, resulting in a lower bulk electrical impedance as the frequency of the applied field is increased.

**Figure 1:**
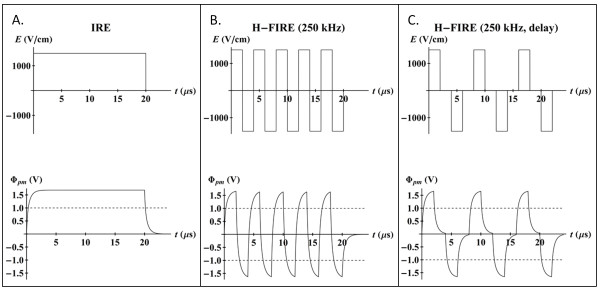
**Characteristic waveforms of IRE and H-FIRE with the corresponding TMP development across the plasma membrane (Φ_*pm*_)**. The 1500 V/cm unipolar pulse (A) causes the TMP to rise above the critical threshold for IRE (1 V, dashed line). The 1500 V/cm bipolar burst without a delay (B) and with a delay (C) causes the TMP to oscillate around the same critical threshold.

One aspect of the current work was to build on our theoretical results and investigate the *in vivo *potential of high-frequency, bipolar bursts to kill tissue through IRE. As opposed to traditional IRE that uses unipolar pulses (Figure 1A) delivered at a repetition rate of one pulse per second, we postulate that IRE can be achieved with a series of high-frequency, bipolar bursts (Figure 1B and 1C) delivered at the same repetition rate. To the best of our knowledge, these types of waveforms have not been investigated for therapeutic IRE applications. Here, we demonstrate lesion development in a rat brain model at center frequencies up to 500 kHz, which maintain the potential to overcome impedance barriers posed by epithelial layers [20]. Similar waveforms have been studied *in vitro *for reversible applications of electroporation, and were shown to counterbalance both electrolytic reactions for the prevention of electrode breakdown [21] and asymmetrical electroporation caused by the resting TMP [22]. Additionally, for a constant electric field applied to cell suspensions or monolayers, as the center frequency of the squarewave is increased up to 1 MHz (duration of single polarity equal to 500 ns), cell death decreases [23]. We explore this trend *in silico *with an analytical model that predicts the TMP in response to an arbitrary electric field and *in vivo *with the treatment of brain tissue using high-frequency IRE (H-FIRE) with bipolar bursts at frequencies of 250 kHz and 500 kHz.

In conjunction with investigating the ability of H-FIRE to kill tissue, this work serves to evaluate its potential for reducing nerve stimulation. Currently, clinical applications of IRE require the administration of general anesthesia and paralytic agents in order to eliminate muscle contractions during each pulse [24]. In some cases, without a sufficient dose of the paralytic agent, muscle contractions are still visible [3]. Muscle contractions may affect the location of implanted needle electrodes, which can invalidate treatment planning algorithms and prove harmful in treatments near vital structures. The threshold for nerve stimulation increases as the center frequency of bipolar waveforms is increased [25]. In this study, an accelerometer based recording system was used to quantify muscle contractions during conventional IRE with unipolar pulses and H-FIRE at frequencies of 250 kHz and 500 kHz. Our results indicate that H-FIRE can non-thermally ablate tissue without causing muscle contractions. The non-thermal nature of the treatment is confirmed through both histological comparison to IRE controls and the development of a finite element model (FEM) for evaluating potential thermal damage in H-FIRE therapy of the brain.

## Methods

### Analytical Modeling of Transmembrane Potential

TMP development in response to a high-frequency electric field has been described in detail for a spherical cell with an organelle by Kotnik and Miklavčič [26]. Here we extend this model to include squarewave bursts with a delay between the positive polarity and negative polarity pulses. This delay was implemented experimentally as a protective measure for the MOSFET based pulse generation system described below. Under the assumption that cells can be represented by dielectric shells containing and surrounded by a conductive medium, the spatial distribution of electric potential (Φ) in a uniform electric field is described by the general solution to the Laplace equation for an arbitrary number of concentric shells [27]:

(1) Φ(r,θ)=Air+Bir2 cos(θ)

where *r *is the distance from the origin to a point on the cell, *θ *is the angle between the electric field and a point on the cell, and *A *and *B *are constants specific to different cellular regions (*i*), including the extracellular space (*e*), plasma membrane (*pm*), cytoplasm (*c*), nuclear envelope (*ne*), and nucleoplasm (*n*). The constants are determined by solving the boundary conditions of continuity of electric potential (Eq. 2, 3, 4, 5) and electric current density (Eq. 6, 7, 8, 9), in addition to the assumptions of a finite field at *r *= 0 and a uniform field at *r *= ∞. In order to accurately predict the TMP in a time-varying electric field, the admittivity operator (Λ*_i _*= *σ_i _*+ *ε_i _*(∂/∂*t*)) is used in the formulation for the boundary conditions [28]:

(2) $$\Phi_{n}\left(R_{n}-d_{ne},\theta \right)=\Phi_{ne}\left(R_{n}-d_{ne},\theta \right)$$

(3) $$\Phi_{ne}\left(R_{n},\theta \right)=\Phi_{c}\left(R_{n},\theta \right)$$

(4) $$\Phi_{c}\left(R_{c}-d_{pm},\theta \right)=\Phi_{pm}\left(R_{c}-d_{pm},\theta \right)$$

(5) $$\Phi_{pm}\left(R_{c},\theta \right)=\Phi_{e}\left(R_{c},\theta \right)$$

(6) $$\Lambda_{n}{\left(\frac{\partial \Phi_{n}}{\partial r}\right|}_{R_{n}^{}-d_{ne}}=\Lambda_{ne}{\left(\frac{\partial \Phi_{ne}}{\partial r}\right|}_{R_{n}^{}-d_{ne}}$$

(7) $$\Lambda_{ne}{\left(\frac{\partial \Phi_{ne}}{\partial r}\right|}_{R_{n}^{}}=\Lambda_{c}{\left(\frac{\partial \Phi_{c}}{\partial r}\right|}_{R_{n}^{}}$$

(8) $$\Lambda_{c}{\left(\frac{\partial \Phi_{c}}{\partial r}\right|}_{R_{c}^{}-d_{pm}}=\Lambda_{pm}{\left(\frac{\partial \Phi_{pm}}{\partial r}\right|}_{R_{c}^{}-d_{pm}}$$

(9) $$\Lambda_{pm}{\left(\frac{\partial \Phi_{pm}}{\partial r}\right|}_{R_{c}^{}}=\Lambda_{e}{\left(\frac{\partial \Phi_{e}}{\partial r}\right|}_{R_{c}^{}}$$

where *d *represents membrane thickness, *R *represents radius, and *ε *and *σ *in the admittivity operator are the dielectric permittivity and conductivity, respectively, of the different cellular regions (Table 1). To avoid working with differential operators, the admittivity operator is transformed into the frequency domain (Λ*_i _*= *σ_i _*+ *sε_i _*), where *s *is the complex frequency (*jω*) of the applied field. The resulting solutions for TMP (ΔΦ) across the plasma membrane and nuclear envelope can then be expressed as [28]:

**Table 1:** Parameter values for TMP simulation

Quantity	Parameter	Value	Units	Reference
Conductivity	*σ_e_*	1.2	S m^-1^	[50]
	*σ_pm_*	3 × 10^-7^		[51]
	*σ_c_*	0.3		[52]
	*σ_ne_*	3 × 10^-7^		set equal to *σ_pm_*
	*σ_n_*	0.3		set equal to *σ_c_*

Permittivity	*ε_e_*	6.4 × 10^-10^	A s V^-1 ^m^-1^	[53]
	*ε_pm_*	4.4 × 10^-11^		[51]
	*ε_c_*	6.4 × 10^-10^		[53]
	*ε_ne_*	4.4 × 10^-11^		set equal to *ε_pm_*
	*ε_n_*	6.4 × 10^-10^		[53]

Radius	*R_c_*	7.5 × 10^-6^	m	estimated from [54]
	*R_n_*	5 × 10^-6^		estimated from [54]

Thickness	*d_pm_*	5 × 10^-9^	m	[55]
	*d_ne_*	5 × 10^-9^		set equal to [55]

(10) $$\Delta \Phi_{pm}\left(s\right)=\Phi_{e}\left(R_{c},\theta \right)-\Phi_{c}\left(R_{c}-d_{pm},\theta \right)=H_{pm}\left(s\right)\;E\left(s\right)\;\cos\left(\theta \right)$$

(11) $$\Delta \Phi_{ne}\left(s\right)=\Phi_{c}\left(R_{n},\theta \right)-\Phi_{n}\left(R_{n}-d_{ne},\theta \right)=H_{ne}\left(s\right)\;E\left(s\right)\;\cos\left(\theta \right)$$

where *H_pm _*(*s*) and *H_ne _*(*s*) are transfer functions that reflect the geometric and dielectric properties of the various cellular regions, the exact formulations of which are given in detail in [26]. *E *(*s*) was obtained by taking the Laplace transform (ℒ) of the time-varying electric field, which was composed of a series of step functions. Equations 10 and 11 were solved in Wolfram Mathematica 8.0 (Champaign, IL, USA), and the inverse Laplace Transform (ℒ^-1^) was taken to produce the time course of TMP in response to bipolar pulses with various frequencies.

### Numerical Modeling of Temperature and Thermal Damage

A 3D finite element model (FEM) for calculating the temperature and potential thermal damage in brain tissue during IRE has been described in detail by Garcia *et al*. [29]. A similar model has been developed here in COMSOL Multiphysics 4.2 (Stockholm, Sweden) for predicting the thermal response to the *in vivo *H-FIRE treatments. Specifically, the electric potential distribution in the tissue was obtained by solving the time-harmonic continuity equation:

(12) $$-\nabla \cdot \left[\left(\sigma +j\omega \varepsilon \right)\nabla \Phi \right]=0$$

where *ω *is the angular frequency of the field. Equation 12 is developed from Maxwell's equations under the electro-quasistatic approximation, which neglects magnetic induction and allows for the expression of the electric field only in terms of electric potential:

(13) E=-∇Φ

Dielectric tissue properties were chosen at 250 kHz to match the center frequency of the waveform delivered experimentally in a majority of the H-FIRE trials. Data generated by Gabriel *et al*. for the conductivity and permittivity of grey matter [30] was interpolated in Mathematica in order to estimate the values at 250 kHz (Table 2). The electro-quasistatic assumption is validated based on the fact that the frequency of the field corresponds to a wavelength (3.5 m) and skin depth (0.5 m) that are greater than the longest dimension in the geometry [31]. The brain subdomain was modeled as a 0.75 cm × 0.75 cm × 0.425 cm ellipsoid having a total volume of 1 cm^3 ^[32]. The maximum applied voltage, electrode dimensions, exposure length, and spacing were modeled to match the *in vivo *configuration described below. Electric potential boundary conditions of Φ = 400 V and Φ = 0 V were applied on the energized and grounded portions of the electrodes, respectively, while the remaining boundaries were treated as electrically insulating (-*n *· *J *= 0), where *J *is the current density. The electrode subdomain was subtracted from the brain subdomain for conservative temperature estimates by preventing any heat dissipative fin effects. A fine mesh was utilized, which consisted of 23989 elements and resulted in less than a 0.5% difference in Joule heating between the electrodes upon further refinements.

**Table 2:** Parameter values for FEM simulation

Physics	Parameter	Value	Units	Reference
Heat conduction	*k*	0.565	W m^-1 ^K^-1^	[56]
	*c_p_*	3680	J kg^-1 ^K^-1^	[56]
	*ρ*	1039	kg m^-3^	[56]

Electric Currents	*σ*	0.145	S m^-1^	[30]
	*ε*	1.95 × 10^-8^	A s V^-1 ^m^-1^	[30]

Damage Processes	*E_a_*	8.033 × 10^5^	J mol^-1^	[33]
	*ζ*	1.676 × 10^129^	s^-1^	calculated from [33,34]

Following a time-harmonic analysis of the electric field distribution, the temperature distribution in the brain was obtained by transiently solving the heat conduction equation with the inclusion of the predetermined Joule heating term (*J *· *E *= (*σ *+ *jωε*)*E *· *E*) as a heat source:

(14) $$\frac{\partial T}{\partial t}=\frac{1}{\rho \;c_{p}}\left[\nabla \cdot \left(k\nabla T\right)+\left(J\cdot E\right)\right]$$

where *c_p _*is the specific heat capacity at constant pressure, *k *is the thermal conductivity, and *ρ *is the density of the tissue (Table 2). Terms associated with the Pennes' Bioheat equation that account for blood perfusion and metabolism commonly used to assess tissue heating during IRE have been neglected for conservative temperature estimates [29]. Additionally, it was assumed that all subdomains were initially at physiologic temperature (37°C) and all boundaries were thermally insulating (-*n *· (-*k *∇*T*) = 0).

Three different methods were evaluated for calculating Joule heating to determine which method was computationally efficient while generating accurate solutions during a 10 s test treatment. One method simulated ten IRE pulses (200 μs duration) at a repetition rate of one pulse per second. Another method modeled ten, 250 kHz H-FIRE bursts (200 μs on-time) at a repetition rate of one burst per second. In the last method, voltage was held constant, but the Joule heating term was scaled by the duty cycle of the burst (200 × 10^-6^) for a 10 s simulation. The parameters chosen had identical energized times, ensuring that equal quantities of energy were delivered to the tissue independent of the method used. The scaled Joule heating approach was eventually adapted for predicting the temperature increase during the entire H-FIRE protocol implemented experimentally, due to the slightly conservative yet accurate temperature distributions that were calculated in a fraction of the simulation time of the other two methods (data not shown). This technique also eliminates the need for an adaptive time stepping algorithm required to resolve microsecond order pulses that occur on the order of seconds. To account for the experimental condition of 180 bursts delivered at a rate of one per second, the Joule heating term was multiplied by the duty cycle (200 × 10^-6^) and the simulation was run for 180 s.

Based on the temperature distribution, the extent of potential thermal damage in the brain was quantified at each time step using the Arrhenius equation:

(15) $$\text{Ω}\left(t\right)=\int \zeta e^{-E_{a}/\left(R\;T\left(t\right)\right)}dt=\int F\;dt$$

where *ζ *is the frequency factor, *E_a _*is the activation energy, *R *is the universal gas constant, and *T*(*t*) is the temperature distribution for a given time (*t*). Due to a lack of data in the literature on Arrhenius parameters for healthy brain, parameters were chosen from glioblastoma cells in the temperature range 40°C to 45°C [33] (Table 2). It is important to note that only *E_a _*is presented in [33], and *ζ *was calculated based on a linear relationship between *E_a _*and ln(*ζ*) for mammalian cells [34]. Thermal damage can include a variety of processes including cell death, microvascular blood flow stasis, and protein coagulation [35], each of which have different parameter values. Here, we have chosen to model the Arrhenius parameters associated with cell death. In terms of finite element modeling of tissue damage, a damage integral value Ω = 1 corresponds to a 63% probability of cell death at a specific point, and a damage integral value Ω = 4.6 corresponds to 99% probability of cell death at that point [36]. Equation 15 was incorporated into Comsol by adding a general PDE solver mode and writing the forcing function (*F*) in logarithmic form:

(16) $$F=e^{\ln\left(\zeta \right)-E_{a}/\left(R\;T\left(t\right)\right)}$$

in order to prevent abrupt changes in the solver. Additionally, all boundaries in the domain were assumed to be insulating (-*n *· Γ = 0), where Γ is the general flux vector, which was assumed to be zero.

### *In Vivo *Experiments

**Figure 2:**
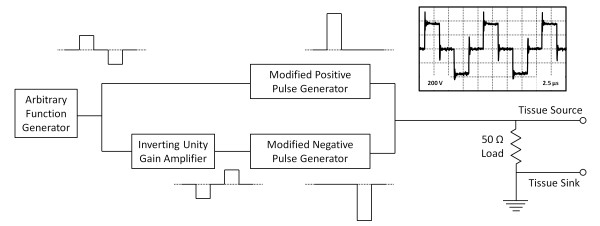
**Schematic diagram of the pulse generation system**. The example output is a portion of the bipolar burst delivered during *in vivo *H-FIRE of the brain.

H-FIRE was performed using a custom pulse generator (Figure 2). Two commercially available monopolar high voltage MOSFET switches (HV1000, Directed Energy, Inc., Fort Collins, CO, USA) were modified so that their outputs would withstand a pulse of opposite polarity. When triggered with a positive 5 V signal, these generators deliver a corresponding positive (HV1000P) or negative (HV1000N) pulse. An unregulated DC power supply was constructed to maintain a sufficient level of charge to deliver 20 A over a 100 μs burst. A center tapped 400 VA transformer (AS-4T320, Antek, Inc., North Arlington, NJ, USA) was rectified and smoothed by a capacitor bank to provide positive and negative power rails to the HV1000P and HV1000N, respectively. The voltage rails were controlled by adjusting the input voltage using a variable transformer, and the maximum output rating of the system was +/- 450 V. For each treatment, an arbitrary function generator (AFG3011, Tektronix, Beaverton, OR, USA) was used to define the parameters of the pulse train to be delivered. A delay equal to the duration of single polarity was included between the pulses in order to protect the MOSFETs from ringing. A unity gain inverting amplifier (AD844, Analog Devices, Norwood, MA, USA) was used to invert this signal and appropriately trigger the negative pulse generator. The outputs of the two monopolar pulse generators were terminated into a 50 Ω load in parallel with the electrodes. This load was used to maintain appropriate pulse characteristics and as a safety to ensure the system was never triggered without an attached load. For comparison, the IRE treatments were performed using the BTX ECM 830 electroporation system (Harvard Apparatus, Holliston, MA, USA).

All study procedures were conducted following Institutional Animal Care and Use Committee approval and performed in a GLP compliant facility. Four, Fischer 344 male rats weighing 200-240 g were anesthetized by intraperitoneal injection of 10 mg/kg xylazine and 60 mg/kg ketamine hydrochloride, and a surgical plane of anesthesia was assessed by loss of the tail pinch reflex. To monitor muscle contractions, a 3-axis accelerometer breakout board (ADXL335, Adafruit Industries, New York, NY, USA) with a sensing range of ± 3 g's was sutured to the dorsum of each rat in the interscapular region at the cervicothoracic junction using 5-0 monocryl suture. Low-pass filter capacitors (0.1 μF) were included at the x, y, and z outputs of the accelerometer for noise reduction. The hair of the skull was clipped and aseptically prepared using povidone-iodine and alcohol solutions. Anesthetized rats were placed in a small animal stereotactic head frame (Model 1350M, David Kopf Instruments, Tungisten, CA, USA). A routine lateral rostrotentorial surgical approach to the skull was made, and 6 mm by 3 mm rectangular parieto-occipital craniectomy defects were created in the right and left aspects of the skull of each rat using a high-speed electric drill. Custom electrodes were inserted into the center of the forelimb area of the sensorimotor cortex of each rat (coordinates relative to Bregma: 1 mm anterior, 2.5 mm lateral, 2 mm dorsoventral) and advanced to a depth of 2 mm beneath the surface of the exposed dura. The electrodes were fashioned by blunting stainless steel acupuncture needles (0.45 mm diameter, Kingli Medical Appliance Co., Wuxi, China) with high grade sandpaper. Exposure length was set to 1 mm by insulating the electrodes with miniature polyimide tubing (25 AWG, Small Parts, Seattle, WA, USA), and the edge-to-edge electrode spacing was set to 1 mm by molding the electrodes in liquid phase polydimethylsiloxane (PDMS) cured in a 10:1 ratio with Sylgard 184 (Dow Corning Corp., Midland, MI, USA) at 150°C for 30 min.

Pulse parameters were chosen based on the results from the analytical and numerical models to ensure the greatest potential for non-thermal tissue ablation. Following electrode insertion, pulses were applied to the right and left cerebral hemispheres, resulting in two treatments per rat (Table 3). All H-FIRE experiments were performed using 180 bursts with a pulse on-time of 200 μs within each burst, and all bursts were delivered at a rate of one per second. In Rat #1 and Rat #2, H-FIRE was applied at voltages of 100 V and 200 V, respectively, to the right hemisphere with a center frequency of 250 kHz (duration of single polarity equal to two microseconds). The left hemisphere of Rat #1 and Rat #2 were treated with 180 IRE pulses (200 μs duration) of equivalent energy. In Rat #3, H-FIRE was applied to the left and right hemispheres at voltages of 300 V and 400 V, respectively, with a frequency of 250 kHz. In Rat #4, H-FIRE was applied at a voltage of 400 V to the right hemisphere with a frequency of 500 kHz (duration of single polarity equal to one microsecond). The left hemisphere of Rat #4 was treated with 90 IRE pulses (200 μs) and an applied voltage of 50 V. This lower energy scenario was designed to compare H-FIRE treatment outcomes to traditional IRE protocols in the brain [37].

**Table 3:** Treatment matrix for *in vivo *experiments

Rat Number	Treatment	Hemisphere	Frequency (kHz)	Voltage (V)
1	IRE	Left	-	100
	H-FIRE	Right	250	100

2	IRE	Left	-	200
	H-FIRE	Right	250	200

3	H-FIRE	Left	250	300
	H-FIRE	Right	250	400

4	IRE	Left	-	50
	H-FIRE	Right	500	400

Immediately following treatment, Rats #3 and #4 were subjected to MRI examinations of the brain while under general anesthesia. The MRI was performed with a 0.2 T MRI scanner using a dual phased array hand/wrist coil for RF signal transmission and reception. Sequence acquisition parameters were as follows: T1-weighted images were acquired using spin echo pulse sequence (TR = 200 ms, TE = 16 ms, FOV = 6 cm, matrix = 256 × 196, slice thickness = 2 mm), and T2-weighted images were acquired using a gradient echo pulse sequence (TR = 3000 ms, TE = 90 ms, FOV = 6 cm, matrix = 256 × 196, slice thickness = 3 mm). T1-weigthed images were obtained following intraperitoneal injection of 0.1 mmol/kg of gadopentetate dimeglumine (Magnevist, Berlex Laboratories, NJ, USA). In all rats, humane euthanasia was performed by cervical dislocation approximately 1 hr post-treatment, and the brain was removed and fixed intact in 10% neutral buffered formalin. Following fixation for 48 hours, an adult rat brain matrix slicer (Zivic Instruments, Pittsburg, PA) was used to obtain contiguous 2 mm coronal brain sections from each animal. Brain and sections were embedded routinely in paraffin, sectioned at 5 μm, and stained with hematoxylin and eosin (H&E).

## Results

### Analytical Modeling of Transmembrane Potential

**Figure 3:**
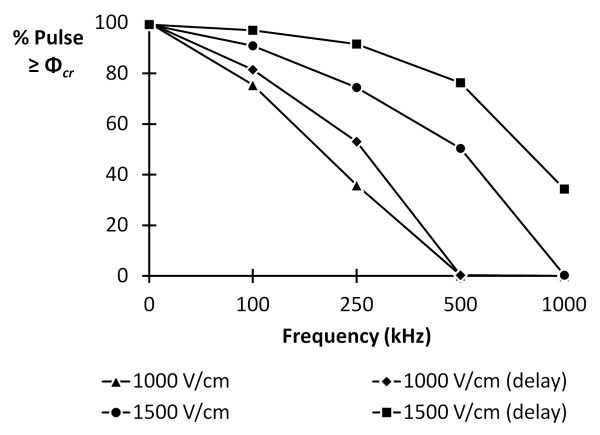
**Comparison of time above the critical threshold (Φ_*cr*_) for IRE at various center frequencies**. Bipolar bursts were simulated with an electric field of 1000 V/cm and 1500 V/cm and an on-time of 20 μs. As the frequency of the applied field is increased, the time above the critical threshold diminishes. This characteristic dispersion is shifted towards higher frequencies for bursts that have a delay between the positive and negative polarity pulses. A conventional IRE pulse is depicted by the 0 kHz data point.

The critical TMP (Φ_*cr*_) across the plasma membrane required to induce IRE is approximately 1 V [38]. This threshold is illustrated in Figure 1 by the dashed, horizontal line on the TMP profiles. All results are presented at the cell pole (*θ *= 0) to show the maximum TMP around the cell. Further, results are only shown for TMP across the plasma membrane, as the TMP across the nuclear envelope never approached the permeabilizing threshold. For an electric field of 1500 V/cm, results indicate that a unipolar pulse (Figure 1A), a 250 kHz bipolar burst (Figure 1B), and 250 kHz bipolar burst that includes delays between the pulses (Figure 1C) are all capable of inducing IRE. However, the time above the threshold TMP varies between the different cases. This is investigated further in Figure 3 for center frequencies of 0, 100, 250, 500, and 1000 kHz, with the 0 kHz case representing the unipolar pulse, and electric fields of 1000 V/cm and 1500 V/cm. The burst width of the bipolar waveform that included delays was twice as long (40 μs) as the corresponding burst with no delays in order to generate an equivalent pulse on-time (20 μs). The amount of time that the TMP was above the critical value was normalized by the on-time and converted to a percentage. Figure 3 illustrates that, for a given frequency, as the electric field is increased from 1000 V/cm to 1500 V/cm, the percentage of the burst above the critical TMP also increases. At 250 kHz, IRE is possible during all waveforms, but at 500 kHz, only the waveforms with amplitudes of 1500 V/cm are capable of inducing IRE. As the center frequency of the burst increases, the percentage of the burst above the critical TMP decreases. However, with the inclusion of delays between the pulses, this characteristic dispersion is shifted towards higher frequencies. At 1 MHz, only the 1500 V/cm waveform with delays can theoretically cause IRE.

### Numerical Modeling of Temperature and Thermal Damage

**Figure 4:**
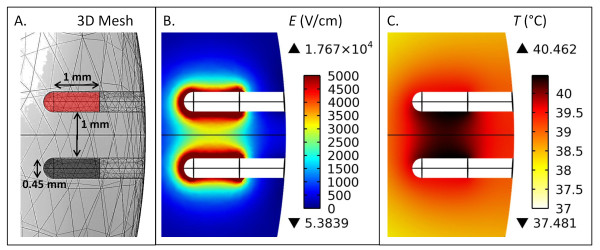
**Schematic diagram of the FEM alongside the predicted electric field (*E*) and temperature (*T*) distributions in brain tissue**. The 3D mesh (A) consisted of 23989 elements. An energized electrode (red) and ground electrode (black) with a 0.45 mm diameter were spaced 1 mm apart (edge-to-edge) and had an exposure length of 1 mm (not including the blunt tip). (B) The resulting electric field distribution in the x-z plane for an applied voltage of 400 V along the energized electrode. (C) The resulting temperature distribution in the x-z plane following the simulation of 180, 200 μs pulses. Upper and lower triangles in the legends depict maximums and minimums within the entire subdomain, respectively.

Results from the simulated H-FIRE treatment of brain tissue are shown in Figure 4. The FEM was developed in 3D, as visualized by the mesh (Figure 4A), and 2D slices were taken in the x-z plane through the center of the brain geometry to display the electric field (Figure 4B) and temperature (Figure 4C) distributions. The electric field decays rapidly with increasing distance from the electrodes, due to their relatively small diameter. The applied voltage-to-distance ratio of 4000 V/cm resulted in a peak electric field of 2979 V/cm along the centerline between the electrodes. Because the electric field relates directly to temperature through the Joule heating term, the application of 180, 200 μs long bursts causes only a 3.5°C increase in temperature near the electrode boundaries. This resulted in a maximum damage integral value of 0.003 at the electrode/tissue interface, which corresponds to a 0.3% probability of cell death from thermal processes. Therefore, all cell death *in vivo *is likely to be a direct result of IRE and not thermal modes.

### *In Vivo *Experiments

**Figure 5:**
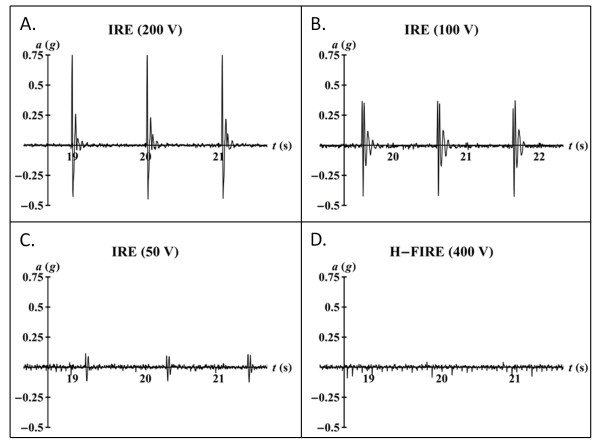
**Snapshot of acceleration (*a*) versus time during IRE and H-FIRE treatments**. Acceleration at the cervicothoracic junction was detected by the accelerometer based recording system during all IRE protocols. None of the H-FIRE protocols resulted in detectable acceleration of the cervicothoracic junction (e.g. shown, 400 V/250 kHz).

**Figure 6:**
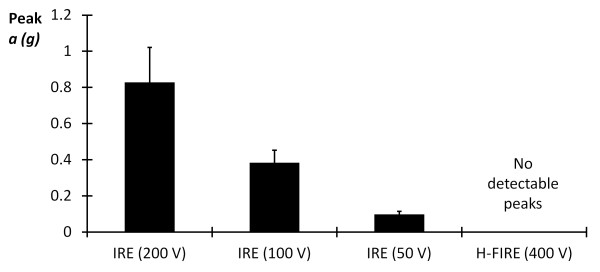
**Peak acceleration (*a*) during IRE protocols averaged over the first 90 pulses**. Mean peak acceleration during IRE treatments at the cervicothoracic junction for each applied voltage was significantly different from each other. H-FIRE resulted in no detectable acceleration of the cervicothoracic junction.

All IRE pulsing protocols were associated with macroscopic muscular contractions of the cervicothoracic junction, which were also palpable to the neurosurgeon, while no visual or tactile evidence of muscular contraction was seen during any of the H-FIRE bursts (Additional File 1). These results were quantitatively confirmed by the data recordings from the accelerometer (Figure 5). Peak acceleration was determined during the first 90 bursts of the highest energy H-FIRE protocol (400 V/250 kHz) and the first 90 pulses of each IRE protocol (50 V, 100 V, 200 V). A one-way ANOVA was used to investigate the effects of each protocol on the ranks of peak acceleration at the cervicothoracic junction. In the event of a significant main effect, pairwise comparisons were completed using Tukey's Honestly Significant Difference (HSD). All statistical analyses were conducted using JMP 7 (Cary, North Carolina, USA) with a significance level of p ≤ 0.05. Results indicate that, even in the highest energy H-FIRE protocol, there are no detectable peaks in acceleration above the inherent noise of the system. However, in all IRE protocols, peaks in acceleration associated with each pulse are detectable above the baseline noise. Further, pairwise comparisons between the various IRE protocols indicated that the mean peak acceleration during each treatment was energy dependent. Specifically, the mean peak acceleration decreased as the applied voltage decreased (Figure 6).

**Figure 7:**
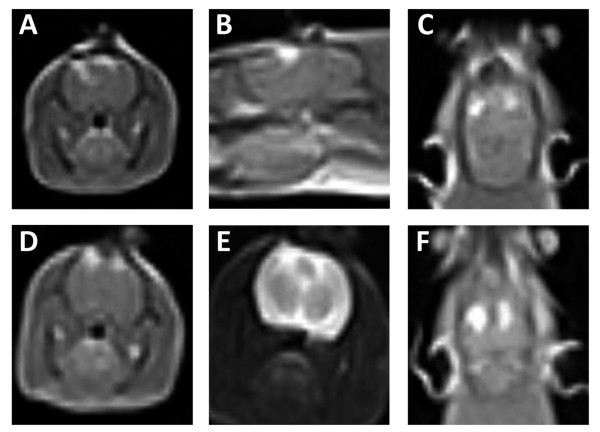
**MRI appearance of H-FIRE and IRE lesions in rat brain**. In all panels, IRE and H-FIRE induced lesions appear as focal hyper-intense regions (white) compared to adjacent untreated cerebrocortical tissue (gray). Top Panels (A-C) obtained from Rat #3, in which both the left and right cerebral hemispheres were treated with H-FIRE at 300 V/250 kHz and 400 V/250 kHz, respectively. Bottom Panels (D-F), Rat #4, which underwent H-FIRE in the right cerebrum at 400 V/500 kHz, and IRE at 50 V in the left cerebrum. Panels A and D, post-gadolinium T1-weighted MRI sequences in the axial plane. Panel B, post-gadolinium T1-weighted MRI sequences in the right parasagittal plane. Panels C and F, post-gadolinium T1-weighted MRI sequences in the dorsal plane. Panel D, T2-weighted MRI sequence in the transverse plane. In all panels, the right side of the brain is on the left side of the panel.

**Figure 8:**
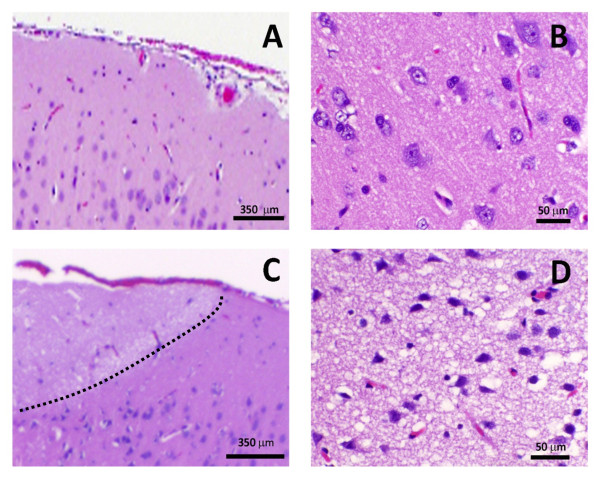
**Histopathology of rat brain tissue**. Untreated (A and B) and H-FIRE treated at 200 V/250 kHz (C and D, Rat #2, right hemisphere). Hematoxylin and eosin stain. The delineation between treated and untreated tissue is shown in Panel C (black, dotted line)

All treatments evaluated in this study produced ablative lesions in brain tissue, as evaluated with MRI examinations (Figure 7) and pathologic preparations (Figure 8). In Rats #3 and #4, the MRI characteristics of both H-FIRE and IRE lesions were similar. Lesions appeared as focal, ovoid to elliptical, T1 iso- to hypo-intense, uniformly and markedly contrast enhanced (Figure 7A, B, C, D, F), and T2 hyper-intense (Figure 7E). All lesions were well demarcated from adjacent, normal brain tissue and appeared similar in size. A comparable size IRE lesion was produced at lower energy, as compared to the H-FIRE lesions. However, as mentioned, IRE even at the lowest energy scenario produced muscle contractions, while the highest energy H-FIRE protocol did not (Figure 6).

**Figure 9:**
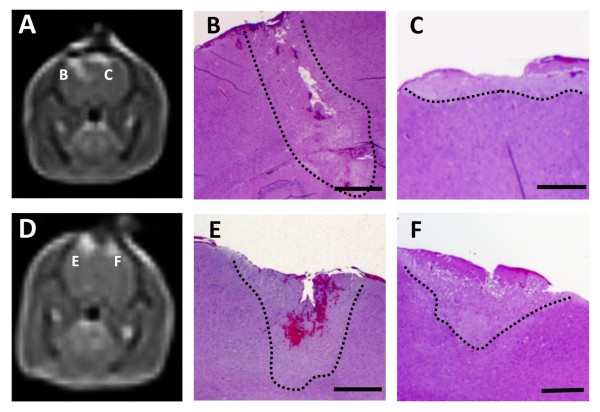
**MRI and corresponding neuropathology of rat brain tissue lesioned with H-FIRE and IRE**. Top Panels (A-C) obtained from Rat #3, in which both the right (Panel B) and left (Panel C) cerebral hemispheres were treated with H-FIRE at 400 V/250 kHz and 300 V/250 kHz, respectively. Bottom Panels (D-F), Rat #4, which underwent H-FIRE in the right cerebrum at 400 V/500 kHz (Panel E), and IRE in the left cerebrum at 50 V (Panel F). Panels A and D are the same as those presented in Figure 7A and D, with an outline of the lesions that are further represented in Panels B, C, E, and F (Hematoxylin and eosin stain, bar = 1 mm). In Panels B, C, E, and F, the delineation between treated and untreated tissue is shown (black, dotted line). In Panels A and D, the right side of the brain is on the left side of the panel.

Compared to untreated brain (Figure 8A and 8B), histopathologic examination of brain sections from all treatments demonstrated clear areas of ablation indicated by pallor of the treated tissue that was sharply delineated from adjacent normal brain (Figure 8C, Figure 9). H-FIRE and IRE lesions were predominantly characterized by areas of complete obliteration of cerebrocortical architecture by an eosinophilic, vacuolated amorphous debris (Figure 8C and 8D). In Rat #1, the H-FIRE ablation zone was confined to regions of elevated electric field surrounding the electrodes, whereas all other pulsing protocols resulted in ablation zones spanning the entire region between the electrodes. Cavitary cerebrocortical defects were induced with H-FIRE in Rat #1 and IRE in Rat #4. Variably sized regions of intraparenchymal hemorrhage were most pronounced immediately adjacent to and within electrode insertion tracks (Figure 9B and 9E). The morphology of remnant neuronal and glial elements within H-FIRE ablated regions demonstrated features of both apoptosis and necrosis, including shrunken and hypereosinophilic cytoplasm, nuclear chromatin condensation, and nuclear pyknosis and karyolysis (Figure 8D). Free glial and neuronal nuclei in various states of degeneration were scattered throughout ablation zones. Inflammation was not a significant feature of IRE or H-FIRE lesions at the time point brains were examined.

## Discussion

The results presented above demonstrate the feasibility of H-FIRE for non-thermally ablating tissue without causing any associated muscle contractions. Specifically, we have shown the ablation of brain tissue by applying bipolar bursts at center frequencies up to 500 kHz. While the pilot data gathered here was not designed to locate an upper limit in terms of frequency at which IRE could still be achieved *in vivo*, the theoretical model of TMP suggests that IRE should be possible up to 1 MHz for an electric field of 1500 V/cm. Interestingly, including a delay between the positive and negative pulses comprising the bipolar burst offers a therapeutic advantage in addition to protecting the MOSFETs in the pulse generation system from ringing. By not forcing a discharge of the TMP with an immediate reversal of polarity, the cell is allowed to return to the resting TMP according to its characteristic time constant. As a result, the TMP is maintained above the critical voltage required for IRE for a longer amount of time. This metric has been recognized before as a potential indicator of treatment outcomes in electroporation based therapies with bipolar waveforms [39].

Due to the small number of observations chosen to assess feasibility, quantitative comparisons between pulse parameters and ablation volumes were not performed. Qualitatively, the fact that IRE was performed with a lower applied voltage in Rat #4 suggests that H-FIRE requires a greater electric field strength than conventional IRE for inducing necrosis, as observed under MRI (Figures 7 and 9). However, even at higher fields, H-FIRE produced no muscle contractions either tactilely, visibly, or through accelerometer recordings at the cervicothoracic junction (Figures 5 and 6). In Rat #1, histopathologic examination revealed that ablation due to H-FIRE occurred only in regions of elevated electric field surrounding the electrodes. This may be explained due to the rapid decline in electric field away from the electrodes predicted by the FEM, and the relatively low percentage of the burst above the critical TMP for the 1000 V/cm waveforms predicted by the analytical model.

The gross histological lesions from the *in vivo *H-FIRE treatments, along with the results of the FEM for predicting the temperature increase in brain tissue during H-FIRE, support the claim that the ablation is not thermally mediated and a direct result of IRE. There is complete uniformity of tissue destruction within targeted H-FIRE areas (Figures 8 and 9), which is in contrast to thermal ablation procedures, such as radiofrequency (RF) ablation [40]. Additionally, H-FIRE results in a sharp transition zone between lesioned and normal brain (on the order of 10-20 μm), which is in agreement with previous intracranial studies on traditional non-thermal IRE [37,41]. The transition zone in RF ablation is characterized by regions of partial tissue damage of anywhere from 100 μm to a few millimeters [42].

Due to the greater electric field strength required to induce IRE with high-frequency, bipolar bursts, evaluating the thermal damage probability in the tissue gains importance in H-FIRE treatment planning. Here, the FEM was conservative in the sense that all boundaries were thermally insulating, no electrodes were present in the subdomain to dissipate heat, and the duty cycle approach was implemented for calculating Joule heating as opposed to simulating individual pulses. The *in vivo *treatments resulted in no predictable thermal damage, due to the small diameter of the electrodes and small exposure length. In the future, temperature predictions should be validated by direct measurements, and the effects of H-FIRE on the electrical conductivity of the tissue should be incorporated into the FEM, as this will have implications for Joule heating. However, it is possible that H-FIRE will produce more subtle changes in tissue conductivity compared to IRE, due to its ability to enhance the capacitive coupling across epithelial layers [20].

The brain was chosen here as a model system due to our expertise and specific interest in advancing electroporation based therapies for the treatment of malignant glioma, including glioblastoma multiforme [43]. Despite attempts to selectively target efferent pathways to the limbs based on electrode placement, all IRE treatment protocols produced contractions of the head, truncal, and limb musculature. Therefore, movement may be caused by direct stimulation of motor regions in the brain, as well as leak currents that directly excite muscle. No hemorrhage occurred, other than an anticipated microhemorrhage along the electrode tracks, and no inadvertent penetration into cerebrospinal fluid pathways occurred, other than traversing of the subarachnoid space at the point of electrode insertion, which might influence leak currents. It is expected that the results obtained in the brain will translate to other tissues, such as the liver, prostate, kidney, and breast, which warrant further investigations. Additionally, studies performed directly in muscle would help elucidate the mechanism responsible for a reduction in nerve stimulation in response to H-FIRE, as the presence of the skull may provide a certain degree of electrical isolation from nearby muscle.

According to classic literature, bipolar waveforms have a higher current threshold for action potential stimulation as compared to monopolar waveforms, which becomes more evident as pulse duration is reduced [25], and bipolar waveforms reduce muscle twitch forces as compared to monopolar waveforms [44]. No electrically induced movement was seen in any of the H-FIRE treatment protocols, ruling out the possibility of tonic contraction. Elimination of patient motion through the delivery of high-frequency, bipolar bursts confers several significant advantages over IRE to both the neurosurgeon and neurosurgical patient that warrants higher energy demands required to achieve ablation. Although electroporation based therapies have proven to be safe and effective in the brain [9,37,43], they require the use of paralytic agents. H-FIRE obviates the need for paralytic agents and, in doing so, provides the possibility for performance of minimally invasive, outpatient intracranial surgery, conscious neurosurgical interventions, procedures in proximity to eloquent areas of the brain, and eliminates the inherent risk associated with general anesthesia. Finally, although not directly evaluated in this study, previous investigators have demonstrated that bipolar pulse delivery at 1 kHz is associated with less patient pain [45].

For frequencies well into the megahertz range, or individual pulse durations on the order of 10-100 nanoseconds, it is possible for a significant amount of current to bypass the plasma membrane through capacitive coupling. As a result, electroporation of both the plasma membrane and intracellular structures can occur for electric field strengths on the order of 10-100 kV/cm [46,47]. These nanosecond pulsed electric fields (nsPEFs) have shown great promise as a cancer therapy due to their ability to induce cell death through apoptotic mechanisms and reduce muscle contractions [48]. One challenge associated with nsPEFs that further distinguishes them from IRE is that the field strength required to induce electroporation of intracellular vesicles and organelles, such as the nucleus, is an order of magnitude greater (40 kV/cm) [49]. This is predominantly due to the small size of vesicles and organelles compared to the overall cell. In H-FIRE, by targeting the plasma membrane, which encompasses the entire cell, the field required to induce cell death is closer in amplitude to IRE protocols than nsPEFs protocols. From an electrical engineering perspective, this simplifies the pulse generation system, and allows for the utilization of silicon based components and commercially available high voltage switches.

## Conclusion

This proof-of-concept study was performed to theoretically and experimentally investigate the potential of high-frequency, bipolar bursts to ablate tissue through IRE and eliminate the associated muscle contractions seen in traditional IRE treatments performed with unipolar pulses. In a rat model, high-frequency IRE (H-FIRE) performed with frequencies up to 500 kHz and voltage-to-distance ratios up to 4000 V/cm produced lesions in brain tissue characteristic of IRE outcomes without causing muscle contractions. Therefore, H-FIRE has the potential to be performed clinically without the administration of paralytic agents, which are used in IRE protocols to mitigate muscle contractions. While not explored explicitly in this study, H-FIRE also offers the benefits associated with charge balancing, including reduced electrolytic contamination. Additionally, high-frequency fields have the potential to overcome impedance barriers posed by low conductivity tissues, which could result in more homogenous and predictable treatment outcomes in heterogeneous systems. Future work should be directed towards further elucidating the relationship between different H-FIRE pulse parameters and ablation volumes, in order to successfully translate this technology for the treatment of tumors. Electroporation based therapies are gaining momentum as viable treatment options for cancer. H-FIRE has the potential to follow suit due to the added benefits that high-frequency, bipolar bursts provide.

## Abbreviations

(IRE): Irreversible Electroporation; (H-FIRE): High-Frequency Irreversible Electroporation; (TMP): Transmembrane Potential; (nsPEF): nanosecond Pulsed Electric Field

## Competing interests

The authors declare that they have no competing interests.

## Authors' contributions

CBA, MBS, JHR, PAG, and RVD designed and performed the experiments and numerical modeling. JLC assisted with the design of the pulse generation system. MNR analyzed the experiments and numerical modeling. All authors read and approved the final manuscript.

## Supplementary Material

Additional file 1 **Video of *in vivo *IRE and H-FIRE experiments**. Click here for file
